# Chinese Herbal Medicine for the Treatment of Obesity-Related Hypertension

**DOI:** 10.1155/2013/757540

**Published:** 2013-06-18

**Authors:** Jie Wang, Bo Feng, Xingjiang Xiong

**Affiliations:** Department of Cardiology, Guang'anmen Hospital, China Academy of Chinese Medical Sciences, Beixiange No. 5, Xicheng District, Beijing 100053, China

## Abstract

*Objectives*. To assess the clinical evidence of Chinese herbal medicine (CHM) for obesity-related hypertension. Search Strategy. Electronic databases were searched until January, 2013. *Inclusion Criteria*. We included randomized clinical trials (RCTs) testing CHM against nondrug therapy and conventional western medicine, or combined with conventional western medicine against conventional western medicine. *Data Extraction and Analyses*. Study selection, data extraction, quality assessment, and data analyses were conducted according to Cochrane standards. *Results*. 11 trials were included. Methodological quality was evaluated as low. 1 trial investigated the efficacy of CHM plus nondrug therapy versus nondrug therapy. Positive results in diastolic blood pressure (DBP) (WMD: −5.40 [−5.88, −4.92]; *P* < 0.00001) were found in combination group. 1 trial investigated the efficacy of CHM versus conventional western medicine. Positive results in systolic blood pressure (SBP) (WMD: −1.39 [−2.11, −0.67]; *P* = 0.0002) were found in CHM. 9 trials investigated the efficacy of CHM plus conventional western medicine versus conventional western medicine. Positive results in SBP (WMD: -6.71 [−11.08, −1.25]; *P* = 0.02) were found in combination group. The safety of CHM is unknown. *Conclusions*. No definite conclusion could be got due to poor methodological quality. Rigorously designed trials are warranted to confirm these results.

## 1. Introduction

Obesity has become an increasingly important medical problem, which is one of the most prevalent nutritional disorders soaring in industrialized countries and progressively increasing in the developing world [1, 2]. Obesity-related hypertension in patients is becoming more prevalent around the world [3, 4]. The number of English language citations in PubMed for “obesity AND hypertension” progressively increased from 203 in 1990 to 1, 427 in 2009, with most of the increase occurring in the past decade [5]. The putative physiologic mechanisms of obesity-related hypertension are complex. A combination of factors, including activation of renin-angiotensin-aldosterone system (RAAS), endothelial dysfunction, oxidative and inflammatory stress, sodium and fluid retention, decrease of sensitivity to natriuretic peptides, and insulin resistance, may contribute to obesity-related hypertension [6–8]. In addition, high plasma leptin concentration and increased sympathetic nervous system (SNS) activity have close relationship with elevation of blood pressure (BP), which may be an important mechanism in obesity-related hypertension [9, 10]. The major goal of obesity-related hypertension treatment is the prevention of obesity and its complications, including hypertension. Currently, treatment programs include nonpharmacologic treatment (weight loss, dietary calorie restriction, exercise, and increased physical activity) and pharmacologic treatment (drugs promoting weight loss, antihypertensive drugs) aiming at reducing weight in the obese and BP [11].

Currently, the prevention and management of obesity-related hypertension are the major public health challenges. However, due to the complex pathological mechanisms of obesity-related hypertension, the medication effects have proved to be far from satisfactory. Many of the pharmaceuticals have unwanted side effects, and the potential for tolerance and dependence, which have limited the clinical efficacy to some extent [12, 13]. Effective treatment of obesity-related hypertension is limited by availability, cost, and adverse effects of conventional western medicine (WM) treatment [14]. Thus, due to the limitations and concerns with current available WM treatments, some people, especially in Asia, have turned to complementary and alternative medicine (CAM) [15–19], including traditional Chinese medicine (TCM) [20–22], for lowering BP, weight, and its related symptoms in searching for a treatment modality with potential efficacy and few adverse effects (AEs) both in developed and developing countries [23–26]. 

As we know, with increasing awareness of people's health care, drugs with natural products as raw materials are gradually favored by people all over the world for their unique advantages in preventing and curing diseases, rehabilitation, and health care [27, 28]. As one essential aspect of TCM, Chinese herbal medicine (CHM) is widely used in Southeast Asia including Japan, South Korea, Malaysia, and Vietnam with a 3000-year-old history [29, 30]. Recently, increasing number of clinical trials and systematic reviews (SRs) have been conducted and published, and it showed that CHMs appear to be effective in lowering BP smoothly [31–37]. Until now, a number of clinical trials of CHM for obesity-related hypertension have been conducted and reported with positive findings [38–42]. However, there have been no systematic English literature reviews to examine this modality. The TCM herbs that are thought to exert treatment of obesity-related hypertension effects require the same supportive evidence as modern medicines. Thus, it is necessary to evaluate the beneficial and harmful effects of CHM for obesity-related hypertension in randomized trials. To our knowledge, this is the first systematic English review on CHM for obesity-related hypertension.

## 2. Methods

### 2.1. Database and Search Strategies

Literature available through both English and Chinese search engines that discusses the potential uses of CHM for the treatment of obesity-related hypertension is included for further analysis. Literature searches were conducted in the following 7 electronic databases: Cochrane Library (January, 2013), EMBASE (1980–2013), PubMed (1959–2013), Chinese National Knowledge Infrastructure (CNKI) (1980–2013), Chinese Scientific Journal Database (VIP) (1989–2013), Chinese Biomedical Literature Database (CBM) (1978–2013), and Wanfang data (1998–2013). Reference lists of retrieved papers were also searched. As CHM is mainly practiced and used in China, 4 major databases in Chinese were searched to retrieve the maximum possible number of trials of CHM for obesity-related hypertension. All of those data searches were ended on January 17, 2013. Ongoing registered clinical trials were searched in the website of Chinese clinical trial registry (http://www.chictr.org/) and international clinical trial registry by US national institutes of health (http://clinicaltrials.gov/). The following search terms were used individually or combined: “obesity-related hypertension,” “obesity hypertension,” “traditional Chinese medicine,” “Chinese herbal medicine,” “Chinese herb,” “Chinese medicine,” “herb,” “herbal medicine,” “clinical trial,” and “randomized controlled trial.”

### 2.2. Inclusion Criteria

Only patients with obesity-related hypertension could be involved in this review. Among them, hypertension is diagnosed by systolic blood pressure (SBP) ≥ 140 mmHg and diastolic blood pressure (DBP) ≥ 90 mmHg; obesity is diagnosed by actual weight which exceeds the standard weight by 20%, Body Mass Index (BMI) ≥ 25, and fat percentage (*F*%) ≥ 30. All randomized controlled trials (RCTs) of Chinese herbal medicine used alone versus nondrug therapy (proper diet, exercise, etc.) and conventional western medicine were included. RCTs combined CHM with conventional western medicine versus conventional western medicine were included as well. There were no restrictions on population characteristics, language, and publication type. The main outcome measure was blood pressure. Duplicated publications reporting the same groups of participants were excluded.

### 2.3. Data Extraction and Quality Assessment

Two authors conducted the literature searching (X. J. Xiong, B. Feng), study selection (X. J. Xiong, B. Feng), and data extraction (X. J. Xiong, B. Feng) independently. The extracted data included authors, title of study, year of publication, study size, age and sex of the participants, study characteristics, diagnosis standard, details of methodological information, name and component of Chinese herb formulae, treatment process, details of the intervention and control, outcomes, and adverse effects for each study. Disagreement was resolved by discussion and reached consensus through a third party (J. Wang).

Methodological quality of included trials was assessed independently according to the criteria from the Cochrane Handbook for Systematic Review of Interventions, Version 5.1.0 (X. J. Xiong, B. Feng) [43]. The items included random sequence generation (selection bias), allocation concealment (selection bias), blinding of participants and personnel (performance bias), blinding of outcome assessment (detection bias), incomplete outcome data (attrition bias), selective reporting (reporting bias), and other bias. The quality of all the included trials was categorized to low/unclear/high risk of bias (“Yes” for a low of bias, “No” for a high risk of bias, and “Unclear” otherwise). Then trials were categorized into three levels: low risk of bias (all the items were in low risk of bias), high risk of bias (at least one item was in high risk of bias), and unclear risk of bias (at least one item was in unclear).

### 2.4. Data Synthesis

Revman 5.1 software provided by the Cochrane Collaboration was used for data analyses. Dichotomous data were presented as risk ratio (RR) and continuous outcomes as mean difference (MD), both with 95% confidence interval (CI). Heterogeneity was recognized significant when *I* ^2^ ≥ 50%. Fixed effects model was used if there is no significant heterogeneity of the data; random effects model was used if significant heterogeneity existed (50% < *I* ^2^ < 85%). Publication bias would be explored by funnel plot analysis if sufficient studies were found.

## 3. Result

### 3.1. Description of Included Trials

**Figure 1:**
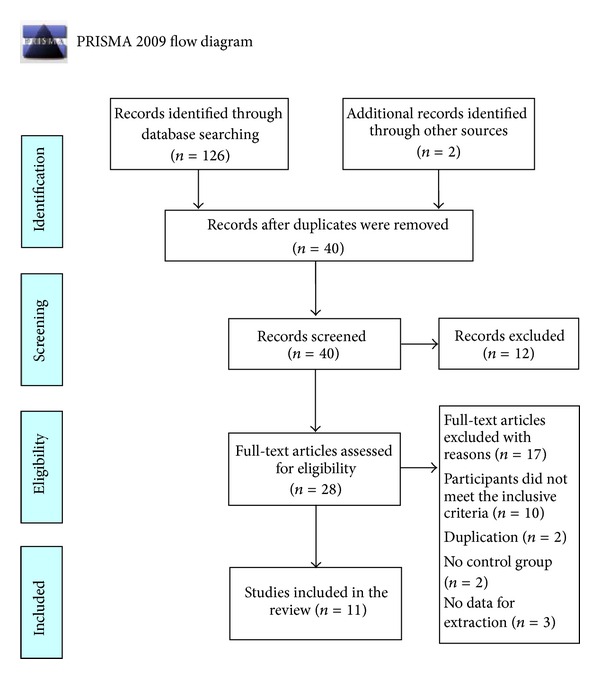
PRISMA 2009 flow diagram.

A flow chart depicted the search process and study selection (as shown in Figure 1). After primary searches from the above 7 electronic databases, 126 articles were retrieved: CNKI (*n* = 60), VIP (*n* = 21), CBM (*n* = 20), Wanfang data (*n* = 19), Cochrane Library (*n* = 1), Pubmed (*n* = 3), and EMBASE (*n* = 2). After primary searches from the databases, 40 articles were screened. After reading the titles and abstracts, 12 articles of them were excluded. Full texts of 28 articles were retrieved, and 17 articles were excluded with reasons listed as follows: participants did not meet the inclusive criteria (*n* = 10), duplication (*n* = 2), no control group (*n* = 2), and no data for extraction (*n* = 3). In the end, 11 clinical trials were included [44–54]. All of these RCTs were conducted in China and published between 2003 and 2012. All of them were published in Chinese. The characteristics of included trials were listed in Table 1.

734 patients with obesity-related hypertension were included. There was a wide variation in the age of subjects (35–76 years). 11 trials used 4 diagnostic criteria of hypertension, 4 trials [44, 45, 53, 54] used Chinese Guidelines for the Management of Hypertension-2005 (CGMH-2005), 2 trials [46, 51] used Chinese Guidelines for the Management of Hypertension-2010 (CGMH-2010), 3 trials [47–49] used 1999 WHO-ISH guidelines for the management of hypertension (1999 WHO-ISH GMH), and 2 trials [50, 52] used 1979 WHO guidelines for the management of hypertension (1979 WHO GMH). 11 trials used 3 diagnostic criteria of obesity, 1 trial [44] used Redefinition and Treatment of Obesity in the Asia Pacific Region-2002 (RTOAPR-2002), 3 trials [45, 47, 48] used Asia Adult Standard of International Obesity by Special Working Group-2000 (AASIOSWG-2000), 4 trials [46, 51, 53, 54] used Prevention and Control Guidelines of Overweight and Obesity in Chinese Adult-2003 (PCGOOCA-2003), and 3 trials [49, 50, 52] only demonstrated patients with obesity (e.g., BMI ≥ 25) without specific information about diagnostic standard. TCM diagnostic criteria were also declared in 5 trials [46, 49, 51, 53, 54]; however, the other 6 trials [44, 45, 47, 48, 50, 52] have not mentioned it at all.

Interventions included CHM used alone or combined with conventional western medicine. The controls included conventional western medicine or nondrug therapy (proper diet, exercise, etc.). CHM included modified Fuling Guizhi Baizhu Gancao decoction [44], modified Liujunzi decoction [45], modified Wendan decoction [46], Pinggan Yishen Ditan decoction [47], Naoxintong capsule [48], decoction of calming liver, expelling phlegm and removing blood stasis [49], Qingre huoxue qutan decoction [50], Shugan yunpi decoction [51], Taohong erchen tianma decoction [52], decoction of activating blood and expelling phlegm [53], and Huanglian Wendan decoction [54]. Among them, 2 kinds of CHM were reviewed, including capsules and herbal decoction as follows: Naoxintong capsule (*n* = 1) [48]; herbal decoction (*n* = 10) [44–47, 49–54]. Among the included clinical trials, 1 trial [44] compared CHM plus nondrug therapy with nondrug therapy; 1 trial [51] compared CHM using alone with conventional western medicine; 9 trials [45–50, 52–54] compared the combination of CHM and conventional western medicine with conventional western medicine. The total treatment duration ranged from 3 to 12 weeks. All of the 11 trials used BP as the main outcome measure. The characteristics of 11 trials were summarized in Table 1. The compositions of different CHM were presented in Table 2. 

### 3.2. Methodological Quality of Included Trials

According to the predefined quality assessment criteria, the methodological quality of the majority of the included 11 trials was assessed to be of general low (as shown in Table 3). All the included trials have mentioned the randomized allocation of participants; however, only 5 trials reported the methods for sequence generation including random number table [44, 45, 51, 53] and drawing [47]. No specific information was provided in the other 6 trials to judge whether or not it was conducted properly [46, 48–50, 52, 54]. Allocation concealment, blinding of participants and personnel, and blinding of outcome assessment were not mentioned in these 11 trials. No trials have reported drop-out and a pretrial estimation of sample size. Also no trials have mentioned the follow-up. We tried to contact with the authors who conducted the trials by telephone, fax, and email for further detailed information mentioned above; however, no information has been provided to date.

### 3.3. Effect of the Interventions

#### 3.3.1. CHM Plus Nondrug Therapy versus Nondrug Therapy

**Figure 2:**
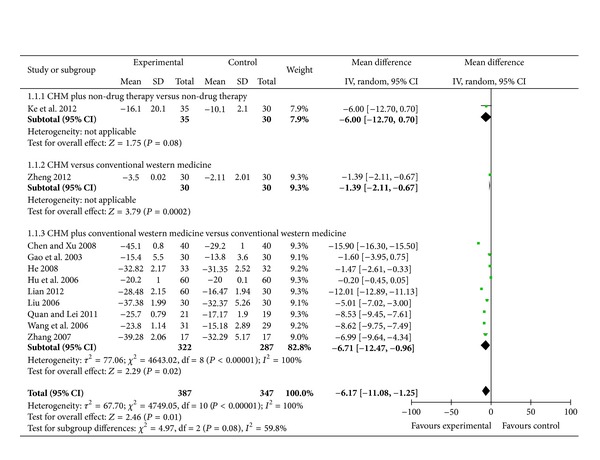
Analyses of systolic blood pressure (SBP).

1 trial investigated the efficacy of CHM plus nondrug therapy for the treatment of obesity-related hypertension as compared to nondrug therapy [44]. When it comes to SBP, meta-analysis showed there is no significant effect of modified Fuling Guizhi Baizhu Gancao decoction plus nondrug therapy as compared to nondrug therapy in SBP (WMD: −6.00 [−12.70, 0.70]; *P* = 0.08) (as shown in Figure 2).

**Figure 3:**
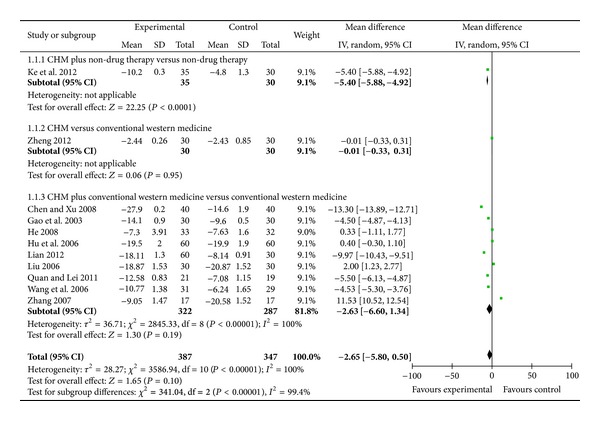
Analyses of diastolic blood pressure (DBP).

When it comes to DBP, meta-analysis showed that there is beneficial effect of modified Fuling Guizhi Baizhu Gancao decoction plus nondrug therapy as compared to nondrug therapy in DBP (WMD: −5.40 [−5.88, −4.92]; *P* < 0.00001) (as shown in Figure 3).

#### 3.3.2. CHM versus Conventional Western Medicine

1 trial reported CHM used alone versus conventional western medicine [51]. When it comes to SBP, meta-analysis showed beneficial effect of Shugan yunpi decoction as compared to conventional western medicine in SBP (WMD: −1.39 [−2.11, −0.67]; *P* = 0.0002) (as shown in Figure 2).

When it comes to DBP, meta-analysis showed there is no significant effect of Shugan yunpi decoction as compared to conventional western medicine in DBP (WMD: −0.01 [−0.33, 0.31]; *P* = 0.95) (as shown in Figure 3).

#### 3.3.3. CHM Plus Conventional Western Medicine versus Conventional Western Medicine

A total of 9 trials reported the effect of CHM plus conventional western medicine versus conventional western medicine [45–50, 52–54]. When it comes to SBP, 5 trials demonstrated better effect favoring CHM: modified Liujunzi decoction plus benazepril mildly lowered SBP than benazepril [45]; modified Wendan decoction plus benzenesulfonic levamlodipine significantly lowered SBP than benzenesulfonic levamlodipine [46]; Pinggan Yishen Ditan decoction plus benazepril hydrochloride mildly lowered SBP than benazepril hydrochloride [47]; decoction of activating blood and expelling phlegm plus perindopril mildly lowered SBP than perindopril [53]; and Huanglian Wendan decoction plus amlodipine besylate significantly lowered SBP than amlodipine besylate [54]. Meta-analysis showed beneficial effect on the combination group as compared to conventional western medicine group (WMD: −6.71 [−11.08, −1.25]; *P* = 0.02) (as shown in Figure 2).

When it comes to DBP, 6 trials demonstrated better effect favoring CHM: modified Liujunzi decoction plus benazepril mildly lowered DBP than benazepril [45]; modified Wendan decoction plus benzenesulfonic levamlodipine mildly lowered DBP than benzenesulfonic levamlodipine [46]; Pinggan Yishen Ditan decoction plus benazepril hydrochloride significantly lowered DBP than benazepril hydrochloride [47]; decoction of calming liver, expelling phlegm and removing blood stasis plus nitrendipine mildly lowered DBP than nitrendipine [49]; decoction of activating blood and expelling phlegm plus perindopril mildly lowered DBP than perindopril [53]; and Huanglian Wendan decoction plus amlodipine besylate significantly lowered DBP than amlodipine besylate [54]. Meta-analysis showed no beneficial effect on the combination group as compared to conventional western medicine group (WMD: −2.63 [−5.80, 0.50]; *P* = 0.19) (as shown in Figure 3).

### 3.4. Publication Bias

The number of the included trials was too small to conduct any sufficient additional analysis of publication bias.

### 3.5. Adverse Effect

No trials have reported the outcome of adverse events (AEs).

## 4. Discussion and Perspective

Currently, CHM used alone or combined with conventional western medicine has been widely used as an alternative and effective method for the treatment of obesity-related hypertension in clinical treatment in China. Until now, many clinical studies of formulas used for therapy of obesity-related hypertension verified the clinical effect ranging from case reports and case series to controlled observational studies and randomized clinical trials. However, there is no critically appraised evidence such as SRs on potential benefits and harms of CHM for obesity-related hypertension to justify their clinical use and their recommendation. Thus, this paper aims to assess the current clinical evidence of the frequently used CHM for obesity-related hypertension. To our knowledge, this is the first systematic English review of the role of Chinese herbs in the treatment of obesity-related hypertension.

This systematic review included 11 randomized trials and a total of 734 participants. In this review, some CHMs have demonstrated potential positive effect for obesity-related hypertension on either SBP or DBP. As compared to nondrug therapy group, positive results in DBP (WMD: −5.40 [−5.88, −4.92]; *P* < 0.00001) were found about CHM plus nondrug therapy group, indicating that DBP could be decreased by 5.40 mmHg after the combination therapy treatment. As compared to conventional western medicine group, positive results in SBP (WMD: −1.39 [−2.11, −0.67]; *P* = 0.0002) were found about CHM group, indicating that SBP could be decreased by 1.39 mmHg after CHM treatment. As compared to conventional western medicine group, positive results in SBP (WMD: −6.71 [−11.08, −1.25]; *P* = 0.02) were found about CHM plus conventional western medicine group, indicating that SBP could be decreased by 6.71 mmHg after combination therapy treatment. However, according to the low methodological qualities and potential publication bias, available data are not adequate enough to draw a definite conclusion of CHM for obesity-related hypertension. What is more, although the statistical data is positive (*P* < 0.05), changes of data, either SBP or DBP, were very slightly, indicating that differences between the treatment group and control group were so small with limited clinical significance. All of these problems and shortcomings will weaken the evidence greatly. And these findings should be interpreted conservatively. Several limitations should be considered as below.

Firstly, the majority of the included trials were assessed to be of general poor methodological quality according to the predefined quality assessment criteria by using criteria from the Cochrane Handbook for Systematic Review of Interventions, Version 5.1.0. All of the included 11 trials in this review had risk of bias in design, methodology, implement, and reporting. Only inadequate reporting of study design, allocation sequence, allocation concealment, double blinding, intention to treat analysis, and drop outs account in the majority of trials was provided. The randomized allocation of participants was mentioned in all trials. However, only 5 trials stated the specific methods for sequence generation including random number table and drawing. The majority of trials only declared that all of the patients with obesity-related hypertension were randomly divided into treatment group and control group without the detailed information. Thus, whether it was conducted rightly or effectively remains doubtful. No trials have conducted allocation concealment. Additionally, as double blinding (blinding of participants and personnel and blinding of outcome assessment) was not mentioned in all trials, the potential performance bias and detection bias would be generated due to patients and researchers being aware of the therapeutic interventions for the subjective outcome measures. None of them have reported drop-out or withdraw. This will undermine the authenticity and reliability of the studies. As none of them had a pretrial estimation of sample size, whether the sample meets the requirements is still unclear. As no trials had mentioned the followup, long-term treatment effect of CHM is still unknown. Moreover, all the included 11 trials were not multicenter, large-scale RCTs which may have resulted in performance bias. Although we had tried to search more detailed information, no information could be got. That is to say, selection bias, performance bias, location bias, and other biases may overestimate the efficacy of CHM. Therefore, confidence in the results obtained from these trials with a lower level of scientific evidence is questionable and each report should be considered only cautiously.

Secondly, publication biases should be also paid attention to. As only articles published in Chinese and conducted in China could be retrieved, a location bias cannot be ruled out. Although we made effort to avoid language bias and location bias in the literature retrieval process, potential publication bias could not be excluded totally. We have conducted extensive searches for unpublished material, but no unpublished “negative” studies were found.

Thirdly, there is a lack of understanding of adverse effects about CHM. As we know, the safety problem is an important guarantee for treatment measures [55, 56]. In this review, all the included trials have not reported the adverse effects. Therefore, a conclusion about the safety of CHM for obesity-related hypertension cannot be made. Maybe it is generally considered that it is safe to use CHM for various diseases in China. However, with increasing reports of the liver and kidney toxicity of CHM, the safety of CHM needs to be monitored rigorously and reported appropriately in the future RCTs.

Fourthly, there was a lack of knowledge for the final outcome measures at endpoint [57]. All of the included trials only reported the outcomes such as BP, blood lipids, and symptom improvement. Also no long-term followup was conducted in these trials. Thus, the effect of CHM on the mortality rate or the incidence of complications is still unclear. Future RCTs of CHM with appropriate design need to be carried out to measure the mortality and morbidity of obesity-related hypertension.

Finally, the significant heterogeneity is worthy of being paid attention to. As this review included 11 different Chinese herb formulae for obesity-related hypertension, great heterogeneity will be generated. As a result, it is impossible to conduct any meaningful meta-analysis for a specific Chinese herb or formulae or difficult to undertake subgroup analyses to explore the specific factors that may have an impact on the effects of the treatment regimen. 

In conclusion, no definite clinical evidence of CHM for obesity-related hypertension could be got due to poor methodological quality of including studies. Rigorously designed trials seem to be warranted to confirm the results.

## Figures and Tables

**Table 1:** Characteristics and methodological quality of included studies.

Study ID	Sample	Diagnosis standard	Intervention	Control	Course (week)	Outcome measure
Ke et al. (2012) [44]	65	CGMH-2005; RTOAPR-2002; TCM diagnostic criteria (unclear)	Modified Fuling Guizhi Baizhu Gancao decoction (1 dose/d) + control	Proper diet + exercise	4	BP
Quan and Lei (2011) [45]	40	CGMH-2005; AASIOSWG-2000; TCM diagnostic criteria (unclear)	Modified Liujunzi decoction (1 dose/d) + control	Benazepril (10 mg qd)	8	BP
Lian (2012) [46]	90	CGMH-2010; PCGOOCA-2003; GCRNDTCM	Modified Wendan decoction (400 mL/d) + control	Benzenesulfonic levamlodipine (5 mg qd)	4	BP
Wang et al. (2006) [47]	60	1999 WHO-ISH GMH; AASIOSWG-2000; TCM diagnostic criteria (unclear)	Pinggan Yishen Ditan decoction (1 dose/d) + control	Benazepril hydrochloride (10 mg qd)	8	BP
Hu et al. (2011) [48]	120	1999 WHO-ISH GMH; AASIOSWG-2000; TCM diagnostic criteria (unclear)	Naoxintong capsule (0.8 g tid) + control	Amlodipine (2.5 mg qd)	12	BP
Gao (2007) [49]	60	1999 WHO-ISH GMH; diagnostic criteria for obesity (unclear); GCRNDTCM	Decoction of calming liver, expelling phlegm and removing blood stasis (1 dose/d) + control	Nitrendipine (10 mg tid)	4	BP
Zhang et al. (2003) [50]	34	1979 WHO GMH; diagnostic criteria for obesity (unclear); TCM diagnostic criteria (unclear)	Qingre huoxue qutan decoction (1 dose/d) + control	Captopril (12.5–25 mg bid)	12	BP
Zheng (2012) [51]	60	CGMH-2010; PCGOOCA-2003; GCRNDTCM	Shugan yunpi decoction (600 mL/d)	Metformin (250 mg tid)	8	BP
Liu (2006) [52]	60	1979 WHO GMH; diagnostic criteria for obesity (unclear); TCM diagnostic criteria (unclear)	Taohong erchen tianma decoction (1 dose/d) + control	Captopril (12.5–25 mg bid)	12	BP
He (2008) [53]	65	CGMH-2005; PCGOOCA-2003; GCRNDTCM	Decoction of activating blood and expelling phlegm (1 dose/d) + control	Perindopril (4 mg qd)	3	BP
Chen and Xu (2008) [54]	80	CGMH-2005; PCGOOCA-2003; GCRNDTCM	Huanglian Wendan decoction (200 mL/d) + control	Amlodipine besylate (5 mg qd)	4	BP

**Table 2:** Composition of formula in the treatment group.

Study ID	Formula	Composition of formula
Ke et al. (2012) [44]	Modified Fuling Guizhi Baizhu Gancao decoction	Fuling (*Poria cocos*) 30 g, Guizhi (cassia twig) 12 g, Baizhu (*Atractylodes*) 30 g, Dangshen (*lanceolata*) 30 g, Dahuang (*Rheum officinale*) 6 g, Yinchen (*Artemisia capillaris* Thunb) 30 g, and Gancao (*Glycyrrhiza*) 10 g

Quan and Lei (2011) [45]	Modified Liujunzi decoction	Dangshen (*lanceolata*) 12 g, Baizhu (*Atractylodes*) 15 g, Fuling (*Poria cocos*) 12 g, Gancao (*Glycyrrhiza*) 6 g, Banxia (*Pinellia ternata*) 12 g, Chenpi (tangerine peel) 12 g, Tianma (*Gastrodia elata*) 12 g, Gouteng (*Uncaria*) 10 g, Xiakucao (*Prunella vulgaris*) 15 g, Juemingzi (cassia seed) 10 g, Shanzha (hawthorn) 10 g, Zexie (*Alisma orientalis*) 10 g, and Dilong (*Lumbricus*) 10 g

Lian (2012) [46]	Modified Wendan decoction	Banxia (*Pinellia ternata*) 10 g, Zhuru (bamboo bark) 12 g, Zhishi (*Citrus aurantium*) 12 g, Chenpi (tangerine peel) 12 g, Fuling (*Poria cocos*) 15 g, Gancao (*Glycyrrhiza*) 6 g, Hongzao (red jujube) 15 g, Tianma (*Gastrodia elata*) 10 g, Gouteng (*Uncaria*) 20 g, and Xiakucao (*Prunella vulgaris*) 15 g

Wang et al. (2006) [47]	Pinggan Yishen Ditan Yin	Tianma (*Gastrodia elata*) 12 g, Gouteng (*Uncaria*) 9 g, Zexie (*Alisma orientalis*) 15 g, Niuxi (Achyranthes root) 20 g, Baizhu (*Atractylodes*) 15 g, Haizao (seaweed) 12 g, Juemingzi (cassia seed) 30 g, Sangjisheng (*Loranthus parasiticus*) 15 g, Dilong (*Lumbricus*) 6 g, Banxia (*Pinellia ternata*) 9 g, and Xiakucao (*Prunella vulgaris*) 12 g

Hu et al. (2011) [48]	Naoxintong capsule	Huangqi (*Astragalus*) 66 g, Chishao (red peony root) 27 g, Danshen (*Salvia miltiorrhiza*) 27 g, Danggui (*Angelica sinensis*) 27 g, Chuanxiong (*Ligusticum chuanxiong* Hort) 27 g, Taoren (peach kernel) 27 g, Honghua (safflower) 13 g, Ruxiang (olibanum) 13 g, Moyao (Myrrh) 13 g, Jixueteng (*Spatholobus suberectus* Dunn) 20 g, Niuxi (achyranthes root) 27 g, Guizhi (cassia twig) 20 g, Sangzhi (*Ramulus mori*) 27 g, Dilong (*Lumbricus*) 27 g, Quanxie (scorpion) 13 g, and Shuizhi (leech) 27 g

Gao (2007) [49]	Decoction of calming liver, expelling phlegm, and removing blood stasis	Tianma (*Gastrodia elata*) 15 g, Gouteng (*Uncaria*) 25 g, Shijueming (abalone shell) 30 g, Xiakucao (*Prunella vulgaris*) 25 g, Fuling (*Poria cocos*) 20 g, Zhuling (*Grifola umbellata*) 20 g, Zexie (*Alisma orientalis*) 15 g, Dafupi (areca peel) 25 g, Dahuang (*Rheum officinale*) 3 g, Danshen (*Salvia miltiorrhiza*) 20 g, Shanzha (hawthorn) 15 g, Jianghuang (*Curcuma longa*) 15 g, and Huangqi (*Astragalus*) 30 g

Zhang et al. (2003) [50]	Qingre huoxue Qutan decoction	Danshen (*Salvia miltiorrhiza*) 12 g, Gouteng (*Uncaria*) 9 g, Shanzha (hawthorn) 15 g, Taoren (peach kernel) 9 g, Zexie (*Alisma orientalis*) 9 g, Dahuang (*Rheum officinale*) 6 g, Huanglian (*Coptis chinensis*) 6 g, Banxia (*Pinellia ternata*) 9 g, Chenpi (tangerine peel) 9 g, Dannanxing (*Pinellia pedatisecta*) 9 g, Fuling (*Poria cocos*) 9 g, and Tianhuafen (trichosanthin) 9 g

Zheng (2012) [51]	Shugan yunpi decoction	Chaihu (*Bupleurum*) 12 g, Cangzhu (rhizoma atractylodes) 12 g, Zhishi (*Citrus aurantium*) 9 g, Houpu (*Magnolia officinalis*) 9 g, Baishao (white peony root) 12 g, Chenpi (tangerine peel) 6 g, Gouteng (*Uncaria*) 30 g, Huanglian (*Coptis chinensis*) 9 g, and Gancao (*Glycyrrhiza*) 6 g

Liu (2006) [52]	Taohong erchen tianma decoction	Taoren (peach kernel) 10 g, Honghua (safflower) 10 g, Chenpi (tangerine peel) 20 g, Banxia (*Pinellia ternata*) 15 g, Fuling (*Poria cocos*) 20 g, Dannanxing (*Pinellia pedatisecta*) 15 g, Shanzha (hawthorn) 20 g, Huanglian (*Coptis chinensis*) 15 g, Danshen (*Salvia miltiorrhiza*) 15 g, Chishao (red peony root) 15 g, Tianma (*Gastrodia elata*) 20 g, and Gouteng (*Uncaria*) 20 g

He (2008) [53]	Decoction of activating blood and expelling phlegm	Banxia (*Pinellia ternata*), Zhuru (bamboo bark), Chenpi (tangerine peel), Tianma (*Gastrodia elata*), Chishao (red peony root), Fuling (*Poria cocos*), Zhishi (*Citrus aurantium*), Zexie (*Alisma*), Shanzha (hawthorn), Huanglian (*Coptis chinensis*), Danshen (*Salvia miltiorrhiza*), and Gancao (*Glycyrrhiza*)*

Chen and Xu (2008) [54]	Huanglian Wendan decoction	Huanglian (*Coptis chinensis*) 15 g, Banxia (*Pinellia ternata*) 10 g, Zhuru (bamboo bark) 10 g, Zhishi (*Citrus aurantium*) 15 g, Chenpi (tangerine peel) 9 g, Gancao (*Glycyrrhiza*) 6 g, Fuling (*Poria cocos*) 15 g, and Hongzao (red jujube) 10 g

*No detailed information could be got about the dosage.

**Table 3:** Quality assessment of included randomized controlled trials.

Included trials	Random sequence generation	Allocation concealment	Blinding of participants and personnel	Blinding of outcome assessment	Incomplete outcome data	Selective reporting	Other sources of bias	Risk of bias
Ke et al. (2012) [44]	Table of random number	Unclear	Unclear	Unclear	Yes	No	Unclear	Unclear
Quan and Lei (2011) [45]	Table of random number	Unclear	Unclear	Unclear	Yes	No	Unclear	Unclear
Lian (2012) [46]	Unclear	Unclear	Unclear	Unclear	Yes	No	Unclear	High
Wang et al. (2006) [47]	Drawing	Unclear	Unclear	Unclear	Yes	No	Unclear	High
Hu et al. (2011) [48]	Unclear	Unclear	Unclear	Unclear	Yes	No	Unclear	High
Gao (2007) [49]	Unclear	Unclear	Unclear	Unclear	Yes	No	Unclear	High
Zhang et al. (2003) [50]	Unclear	Unclear	Unclear	Unclear	Yes	No	Unclear	High
Zheng (2012) [51]	Table of random number	Unclear	Unclear	Unclear	Yes	No	Unclear	Unclear
Liu (2006) [52]	Unclear	Unclear	Unclear	Unclear	Yes	No	Unclear	High
He (2008) [53]	Table of random number	Unclear	Unclear	Unclear	Yes	No	Unclear	Unclear
Chen and Xu (2008) [54]	Unclear	Unclear	Unclear	Unclear	Yes	No	Unclear	High
